# From Multi‐Omics to Visualization and Beyond: Bridging Micro and Macro Insights in CAR‐T Cell Therapy

**DOI:** 10.1002/advs.202501095

**Published:** 2025-05-11

**Authors:** Yuting Gong, Peng Fei, Yicheng Zhang, Yang Xu, Jia Wei

**Affiliations:** ^1^ Department of Hematology Tongji Hospital Tongji Medical College Huazhong University of Science and Technology Wuhan Hubei 430030 China; ^2^ Immunotherapy Research Center for Hematologic Diseases of Hubei Province Wuhan Hubei 430030 China; ^3^ School of Optical and Electronic Information‐Wuhan National Laboratory for Optoelectronics Huazhong University of Science and Technology Wuhan Hubei 430074 China; ^4^ Advanced Biomedical Imaging Facility Huazhong University of Science and Technology Wuhan Hubei 430074 China; ^5^ Key Laboratory of Organ Transplantation Ministry of Education NHC Key Laboratory of Organ Transplantation Key Laboratory of Organ Transplantation Chinese Academy of Medical Sciences Wuhan Hubei 430030 China; ^6^ National Clinical Research Center for Hematologic Diseases Jiangsu Institute of Hematology The First Affiliated Hospital of Soochow University Suzhou Jiangsu 215006 China; ^7^ Institute of Blood and Marrow Transplantation Soochow University Suzhou Jiangsu 215006 China

**Keywords:** artificial intelligence, CAR‐T therapy, multi‐omics, single cell sequencing, spatial omics, tumor microenvironment, visualization

## Abstract

Chimeric antigen receptor T (CAR‐T) cell therapies, a cornerstone of immunotherapy, have demonstrated remarkable efficacy in treating hematological malignancies and have more recently expanded into applications for solid tumors and autoimmune diseases. Emerging multidimensional profiling technologies offer promising solutions for enhancing CAR‐T efficacy, overcoming resistance, and facilitating the development of novel CAR‐T constructs. The integration of genomics, epigenomics, transcriptomics, proteomics, metabolomics, and microbiomics enables a comprehensive understanding of the intrinsic mechanisms underlying CAR‐T therapy, while single‐cell and spatial omics significantly improve data resolution and analytical depth. Coupled with advances in biomedical engineering, visualization technologies form the foundation for omics data generation by bridging microscopic and macroscopic scales and enabling dynamic, 3D in vivo monitoring of CAR‐T behavior. Artificial intelligence (AI) further supports this framework by enabling the analysis of complex, high‐dimensional datasets. This review highlights recent advances in the integration of multidimensional omics within CAR‐T therapy and explores cutting‐edge developments in visualization technologies and AI applications. The full convergence of multi‐omics, visualization tools, and AI is poised to deliver transformative insights into the mechanisms governing CAR‐T cell therapy.

## Graphical abstract

This review explores how multi‐omics, visualization technologies, and AI drive CAR‐T therapy from the micro to the macro level, from preclinical studies to clinical applications. The convergence of these approaches provides deeper insight into CAR‐T cell mechanisms, aids in structural optimization, and significantly enhances treatment efficacy and safety through advanced imaging and computational analysis.

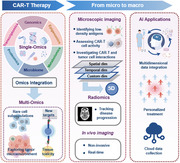

## 1 Introduction

Since the FDA's first approval of CD19 Chimeric antigen receptor T (CAR‐T) cell therapy for acute lymphoblastic leukemia (ALL) in 2017,^[^ ^1^ ^]^ it has become a transformative treatment for hematological malignancies, particularly in large B‐cell lymphoma (LBCL)^[^ ^2^, ^3^ ^]^ and ALL.^[^ ^4^, ^5^ ^]^ Efforts are now underway to extend this therapy to solid tumors,^[^ ^6^, ^7^, ^8^ ^]^ with clinical trials already conducted for various types, including glioblastoma,^[^ ^9^, ^10^ ^]^ neuroblastoma,^[^ ^11^ ^]^ malignant pleural diseases,^[^ ^12^ ^]^ gastric cancer,^[^ ^13^ ^]^ colorectal cancer,^[^ ^14^ ^]^ and pancreatic cancer.^[^ ^15^ ^]^ CAR‐T cell therapy also holds significant potential in the treatment of autoimmune diseases.^[^ ^16^, ^17^, ^18^, ^19^ ^]^ Currently, innovative products such as allogeneic CAR‐T and in vivo CAR‐T therapies continue to emerge.^[^ ^20^, ^21^ ^]^ Despite these advancements, CAR‐T therapy still faces considerable clinical challenges, including off‐target effects, tumor microenvironment (TME)‐mediated suppression,^[^ ^22^ ^]^ and secondary T‐cell malignancies.^[^ ^23^ ^]^ These obstacles underscore the urgent need for safer and more effective CAR‐T products.

The rapid evolution of multi‐omics technologies has provided powerful tools for optimizing CAR‐T therapy. Advances in single‐cell transcriptomics, genomics, proteomics, and epigenomics have yielded new insights into CAR‐T cell biology.^[^ ^24^ ^]^ Single‐nucleus RNA sequencing has addressed limitations associated with frozen sample analysis,^[^ ^25^ ^]^ while single‐cell genomics complements transcriptomics by overcoming the issue of low mutation hotspot coverage.^[^ ^26^ ^]^ In parallel, single‐cell epigenomics has illuminated the epigenetic modifications linked to CAR‐T cell exhaustion.^[^ ^27^ ^]^ Proteomics plays a critical role in evaluating the abundance and functionality of tumor‐associated targets.^[^ ^28^ ^]^ Notably, the advent of spatial transcriptomics has reinvigorated CAR‐T therapy, enabling the precise identification of CAR‐T infiltration sites and activation states within tumors.^[^ ^29^ ^]^ The pivotal role of multi‐omics in advancing CAR‐T therapy has been systematically reviewed.^[^ ^30^, ^31^, ^32^ ^]^

However, multi‐omics research primarily relies on static molecular data, which limits its ability to capture CAR‐T cell dynamics in vivo. Previous reviews have yet to fully recognize the potential of integrating visualization technologies with omics data. The convergence of medical and engineering disciplines has led to groundbreaking advancements in visualization technologies, enabling direct observation of CAR‐T cell structures and real‐time dynamics,^[^ ^33^, ^34^ ^]^ thereby bridging the gap between molecular insights and macroscopic behavior.

Artificial intelligence (AI) is playing an increasingly pivotal role in CAR‐T research. Through deep learning and computer vision, AI significantly enhances the precision of multi‐omics and visualization analyses.^[^ ^35^ ^]^ Additionally, high‐throughput algorithmic platforms have accelerated CAR structure optimization, while robotics, in conjunction with CAR‐T therapy, presents an innovative strategy for precise therapeutic delivery.

This review explores key research priorities in CAR‐T therapy, focusing on recent breakthroughs in multi‐omics integration. It also highlights cutting‐edge advancements in visualization technologies and artificial intelligence, while emphasizing the indispensable role of traditional laboratory techniques. The convergence of multi‐omics, visualization technologies, and AI is poised to offer transformative insights into the mechanisms underlying CAR‐T therapy.

## 2 Bridging Molecular Complexity and Therapeutic Innovation with Multi‐Omics in CAR‐T Cell Therapy

**Figure 1:**
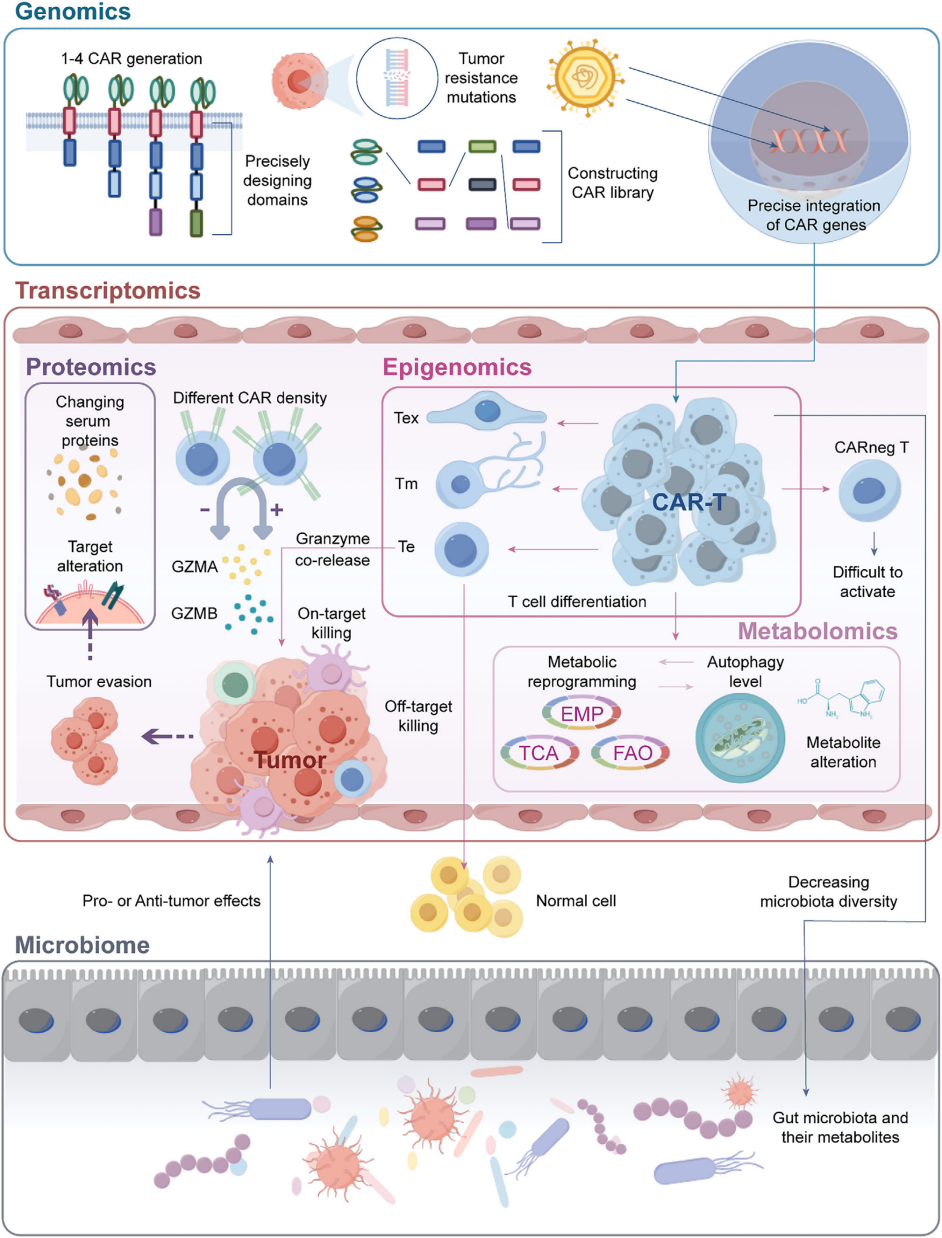
Contributions of distinct omics to advancements throughout the entire process of CAR‐T design, development, and post‐infusion tumor eradication. Genomics focuses on improving CAR‐T structure and gene transduction. Epigenomics centers on CAR‐T cell differentiation and can guide epigenetic reprogramming to enhance therapeutic persistence. Metabolomics explores the relationship between CAR‐T cell metabolism and activity, as well as metabolite changes within the tumor microenvironment. Transcriptomics addresses issues such as toxicity, tumor cytotoxicity, and persistence. Proteomics advances new target discovery and investigates changes in serum proteins. Microbiomics aids in elucidating the interactions between microbiota and CAR‐T cells. Tex: exhausted T cell; Tm: memory T cell; Te: effector T cell; CARneg T: bystander CAR‐T cell; EMP: glycolytic pathway; TCA: tricarboxylic acid cycle; FAO: fatty acid oxidation; GZMA: granzyme A; GZMB: granzyme B.

Distinct analytical approaches from various omics fields provide a multidimensional perspective on CAR T‐cell therapy (**Table** **1**). Genomics, which leverages key technologies such as whole‐genome sequencing (WGS), whole‐exome sequencing (WES), clustered regularly interspaced short palindromic repeat (CRISPR)‐based screens, and personalized cancer profiling by deep sequencing (CAPP‐seq), facilitates CAR‐T cell engineering while elucidating tumor heterogeneity and resistance mechanisms. Epigenomics, which employs whole‐genome bisulfite sequencing (WGBS), an assay for transposase‐accessible chromatin using sequencing (ATAC‐seq), chromatin immunoprecipitation sequencing (ChIP‐seq), and cleavage under targets and mentation (CUT&Tag), uncovers epigenetic regulatory mechanisms that influence CAR‐T cell differentiation and exhaustion. Transcriptomics, including bulk RNA sequencing, single‐cell RNA sequencing (scRNA‐seq), and spatial transcriptomics, provide a comprehensive transcriptional profile of CAR‐T cell function and toxicity. Through single‐cell protein analysis using cytometry with time‐of‐flight (CyTOF), IsoPlexis, quantitative proteomics, and phage immunoprecipitation sequencing (PhIP‐Seq), key therapeutic targets and prognostic markers can be identified. Metabolomics utilizes high‐throughput techniques such as high‐performance liquid chromatography (HPLC) and liquid chromatography‐mass spectrometry (LC‐MS) to investigate metabolic reprogramming in CAR‐T cells and the tumor microenvironment. Additionally, microbiomics, leveraging 16S ribosomal RNA (rRNA) sequencing and metagenomics, has explored the potential influence of the gut microbiome on CAR‐T cell efficacy. Collectively, these multiomics approaches constructed an integrated analytical framework (**Figure** **1**) that offers novel insights for optimizing CAR‐T therapy. The following sections focus on strategies for integrating these omics approaches to address the current challenges in CAR‐T cell therapy.

**Table 1:** Research Focus of Different Omics in CAR‐T Therapy.

Omics	Profiling technology	Research focus in CAR‐T therapy	Advantage	Disadvantage
Genomics	WGS	Design of CAR‐T cell structure	Creation of a comprehensive CAR gene library and acceleration of production	Complexity of data processing and gene annotation
	WES			
	CRISPR screening	Integration of CAR genes into the host cell genome	Facilitates precise design of each CAR structure	Unpredictable gene expression levels and downstream signaling pathways
	CAPP‐seq	Tumor gene alterations during CAR‐T therapy	Clarification of CAR gene integration sites Explanation of tumor heterogeneity	High cost associated with single‐cell genomic analysis
Epigenomics	WGBS ATAC‐seq	Epigenetic reprogramming of CAR‐T cells	Guides of CAR‐T cell proliferation and mitigates exhaustion	Limited insights from epigenomic data alone to identify key regulatory nodes in CAR‐T differentiation
	ChIP‐seq	CAR‐T cell differentiation and exhaustion	Epigenetic engineering of specific CAR‐T cell subsets	
	CUT&Tag			Challenges in data analysis and interpretation
Transcriptomics	scRNA‐seq	Validation of novel CAR‐T cell production methods	Elucidation of downstream signaling pathways and molecular mechanisms	Incomplete representation of protein expression levels from transcriptomic data
	Spatial transcriptomics			
		Understanding various toxicity mechanisms in CAR‐T therapy	Detection of rare cell subsets	
			Tracking cell differentiation over time and space	
		Characterization of relapsed tumor cells		
		Tracing the CAR‐T differentiation process		
		Deepening understanding of CAR‐T cytotoxic mechanisms		
Proteomics	CyTOF	Prediction of tumor targets	Direct research on target specificity	Limited detection capability of current proteomic technologies
	IsoPlexis	Surface proteome changes in asymmetric division and differentiation	Investigation of in vivo environments through plasma protein alterations	
	quantitative proteomics PhIP‐Seq	Role of serum proteomics in prognostic monitoring	Provides a more accurate reflection of protein expression	
Metabolomics	HPLC	Metabolic reprogramming of CAR‐T cells	Detection of metabolic pathways and key enzymes	Requirement for integration of upstream technologies in the downstream analysis process
	LC‐MS			
		Investigation of metabolic changes in the tumor microenvironment and their anti‐tumor effects	Value of metabolite testing for clinical evaluation	
Microbiome	16S rRNA sequencing	Study of the effects of microbiota and its metabolites on CAR‐T cells or tumors	Explains of the interaction between microbiota and CAR‐T therapy	Difficulty in identifying specific microbial species and metabolites
	metagenomic shotgun sequencing	Probiotics‐guided CAR‐T systems	Microorganisms can enhance CAR‐T therapy	Uncertainty surrounding the influence of intratumoral microbiota on CAR‐T therapy

CAR‐T: chimeric antigen receptor t‐cell; DNA: deoxyribonucleic acid; WGS: whole genome sequencing; WES: whole exome sequencing; CRISPR screening: clustered regularly interspaced short palindromic repeats screening; CAPP‐seq: cancer personalized profiling by deep sequencing; WGBS: whole‐genome bisulfite sequencing; ATAC‐seq: assay for transposase‐accessible chromatin with high‐throughput sequencing; ChIP‐seq: chromatin immunoprecipitation sequencing; CUT&Tag: cleavage under targets and tagmentation; scRNA‐seq: single‐cell RNA sequencing; CyTOF: cytometry by time‐of‐flight; PhIP‐Seq: phage immunoprecipitation sequencing; HPLC: high‐performance liquid chromatography; LC‐MS: liquid chromatography‐mass spectrometry.

### 2.1 Structural Optimization and Precision Engineering for CAR‐T Therapy

**Figure 2:**
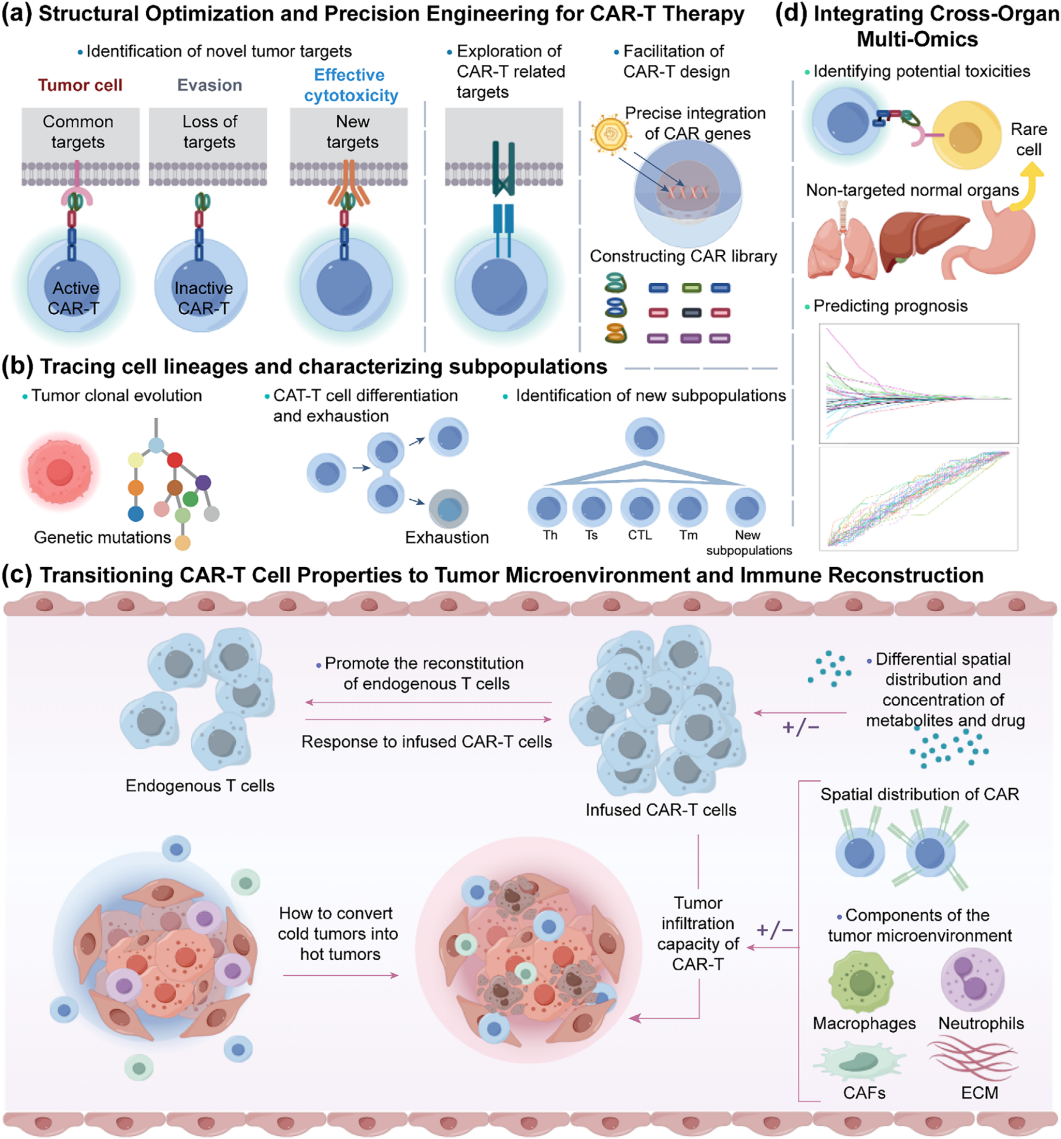
Prospects of multi‐omics analysis in CAR‐T therapy. a) multi‐omics approaches facilitate CAR‐T structural optimization and precision engineering. b) multi‐omics enable cell lineage tracing and the identification of novel cell subsets. c) multi‐omics enhances the understanding of tumor microenvironmental influences on CAR‐T cells. d) multi‐omics aid in assessing tissue toxicity induced by rare cell populations during CAR‐T treatment and predicting patient prognosis. Th: helper T cell; Ts: suppressor T cell; CTL: cytotoxic T lymphocyte; Tm: Memory T cells; CAFs: cancer‐associated fibroblasts; ECM: extracellular matrix.

CAR‐T cell therapy continues to face challenges such as tumor escape, limited cytotoxicity, and reduced persistence. Optimizing CAR‐T cell constructs and engineering enhanced CAR‐T products requires not only the selection of potent, long‐lived T cell subsets but also advancements in CAR design. Target discovery plays a central role in overcoming tumor escape and broadening the therapeutic scope of CAR‐T cells. However, single‐omics approaches often fall short in providing a comprehensive understanding of potential targets. By contrast, integrating multi‐omics data enables systematic identification of novel targets, supports accurate CAR structure design, and facilitates evaluation of functional differences among various constructs (**Figure** **2**a).

The loss of target antigens during tumor evolution is a common cause of relapse following CAR‐T cell therapy. scRNA‐seq has been instrumental in characterizing the transcriptional profiles of relapsed tumors after CAR‐T cell infusion, facilitating the development of multi‐targeted CAR‐T strategies.^[^ ^36^, ^37^, ^38^ ^]^ High‐resolution scRNA‐seq has identified CSF1R and CD86 as potential CAR‐T targets in acute myeloid leukemia (AML)^[^ ^39^ ^]^ and has also revealed candidate targets in multiple myeloma (MM).^[^ ^40^ ^]^ Current research is shifting from scRNA‐seq to spatial transcriptomics to comprehensively map antigen expression within the tumor microenvironment, enabling more precise target identification.^[^ ^41^ ^]^ At the protein level, platforms such as Olink, SomaScan, and mass spectrometry compensate for the limitations of transcriptomics in detecting membrane proteins and have been successfully applied to CAR‐T target discovery in AML,^[^ ^42^, ^43^ ^]^ KMT2A/MLL1‐rearranged B‐ALL,^[^ ^44^ ^]^ glioblastoma,^[^ ^45^ ^]^ and MM.^[^ ^46^, ^47^ ^]^ Although single‐omics analyses have provided valuable insights into tumor biology, they have yet to revolutionize cancer treatment. In contrast, integrative omics approaches, which combine multi‐layered biomarkers, refine molecular classification and enable more accurate tumor diagnosis, thereby advancing the field of precision oncology^[^ ^48^, ^49^ ^]^ Furthermore, integrative multi‐omics analyses have contributed to the development of personalized CAR‐T therapies. For example, a combined WES and RNA‐seq study identified MS4A1 mutations that lead to CD20 loss in relapsed patients,^[^ ^50^ ^]^ providing precise guidance for individualized target selection.

The identification of CAR‐T function‐related targets is essential for improving cytotoxicity and persistence. Genome‐wide CRISPR knockout screening has significantly advanced the discovery of novel targets that influence CAR‐T functionality, including PRDM1,^[^ ^51^ ^]^ RASA2,^[^ ^52^ ^]^ CCNC, and MED12.^[^ ^53^ ^]^ CRISPR‐based scRNA‐seq studies have further revealed that the B‐ and T‐lymphocyte attenuator–herpesvirus entry mediator axis functions as a key immune checkpoint in CAR‐T cell immunotherapy.^[^ ^54^ ^]^ Moreover, coupling CRISPR‐Cas9 with scRNA‐seq enables efficient screening of CAR variants, accelerating structural optimization.^[^ ^55^ ^]^ Standalone scRNA‐seq analyses have also uncovered potential synergistic effects between granzymes A and B, with granzyme A compensating for granzyme B inhibition to maintain tumor cell‐killing capacity,^[^ ^56^ ^]^ suggesting that integrating multiple cytotoxic mechanisms into CAR‐T cells could reduce tumor escape. Metabolomics has revealed additional strategies to enhance CAR‐T cell functionality, such as overexpression of GLUT1,^[^ ^57^ ^]^ IL‐10,^[^ ^6^ ^]^ adenosine deaminase,^[^ ^58^ ^]^ and PRODH2,^[^ ^59^ ^]^ or knockout of IDH2,^[^ ^60^ ^]^ all of which improve antitumor efficacy. Additionally, studies have shown that oxidative stress induced by mitochondrial dysfunction stabilizes HIF‐1α, promoting CAR‐T cell exhaustion, whereas reversing this metabolic reprogramming may alleviate exhaustion.^[^ ^61^ ^]^ Multi‐omics approaches have further identified key regulators of CAR‐T exhaustion. For instance, transcriptomic and proteomic analyses have demonstrated that TIGIT is significantly upregulated in exhausted T cells, and its blockade enhances CAR‐T antitumor efficacy.^[^ ^62^ ^]^ Similarly, CD38 has emerged as a potential biomarker of CAR‐T exhaustion,^[^ ^63^ ^]^ underscoring the value of multi‐omics integration in optimizing CAR‐T cell structure and function.

WGS is critical for evaluating the precise integration of CAR vectors into CAR‐T cells to ensure safety. As CAR‐T therapies become more widely adopted, rare adverse events such as secondary T‐cell lymphomas have attracted increasing attention.^[^ ^64^, ^65^, ^66^ ^]^ Previous genomic profiling studies suggest that these secondary hematological malignancies are more likely linked to pre‐existing malignant clones than to random CAR vector integration.^[^ ^67^, ^68^ ^]^ However, a recent study reported a case where the CAR vector integrated into the SSU72 gene, potentially driving T‐cell malignancy,^[^ ^69^ ^]^ emphasizing the need for WGS‐based safety monitoring. WGS also offers distinct advantages over conventional techniques for assessing off‐target effects associated with gene editing. Notably, a recent study reported the first use of allogeneic CAR‐T therapy for severe myasthenia gravis and systemic sclerosis, further highlighting WGS's role in safety evaluation.^[^ ^70^ ^]^ scRNA‐seq plays a crucial role in assessing the antitumor activity of different CAR constructs^[^ ^71^ ^]^ and, when combined with CAR library screening, accelerates CAR variant optimization.^[^ ^55^ ^]^ Beyond construct design, CAR expression density also influences antitumor efficacy. scRNA‐seq analysis has shown that while high CAR expression enhances cytotoxicity, it also increases the risk of T‐cell exhaustion.^[^ ^72^ ^]^ The optimization of CAR‐T manufacturing processes must therefore align with structural innovations. For example, Iftikhar et al. developed a fusion protein vector targeting CD8α,^[^ ^73^ ^]^ while Charitidis et al. evaluated the efficacy of various viral transduction methods,^[^ ^74^ ^]^ with scRNA‐seq providing essential data to support these technological advancements.

### 2.2 Tracing Cell Lineages and Characterizing Subpopulations

The clonal evolution of tumor cells can contribute to the failure or relapse of CAR‐T therapy. The differentiation potential of CAR‐T cells is closely linked to their ability to resist exhaustion and generate diverse functional subpopulations. Cellular differentiation is accompanied by molecular changes at the DNA, mRNA, and protein levels, which makes it challenging to capture the complete complexity of lineage trajectories using single‐omics approaches. Multiomics integration enables the construction of comprehensive lineage trees, facilitates the identification of tumor resistance mechanisms, and helps define CAR‐T subpopulations with optimal proliferative capacity, persistence, and antitumor activity (Figure 2b). These insights provide a valuable theoretical foundation for optimizing CAR‐T manufacturing.

At the genomic level, WGS plays a pivotal role in elucidating the genomic landscape of tumors in patients who have experienced CAR‐T therapy failure.^[^ ^75^, ^76^, ^77^ ^]^ While WES has a limited capacity to detect non‐coding region variations compared to WGS, its higher sequencing depth allows for a more detailed examination of clonal evolution during CAR‐T treatment.^[^ ^78^, ^79^, ^80^ ^]^ In a pioneering sequential therapy study combining CD7 CAR‐T with allogeneic hematopoietic stem cell transplantation, WES was employed to analyze genetic alterations in patients with CD7‐negative leukemia relapse.^[^ ^81^ ^]^ With optimized next‐generation sequencing (NGS) techniques, WES can further resolve specific gene mutations at single‐cell resolution.^[^ ^82^, ^83^ ^]^ Most genomic studies have focused on mechanisms of tumor antigen loss driving resistance after CAR‐T therapy, while fewer investigations have explored tumor lineage switching.^[^ ^84^ ^]^ For more complex resistance mechanisms, such as antigen masking^[^ ^85^, ^86^ ^]^ and trogocytosis,^[^ ^87^, ^88^ ^]^ genomic studies must be integrated with other omics approaches to elucidate their underlying mechanisms.

At the epigenetic level, WGBS has significantly advanced our understanding of T‐cell differentiation,^[^ ^89^, ^90^ ^]^ alloreactivity,^[^ ^91^ ^]^ and CAR‐T‐cell exhaustion.^[^ ^92^ ^]^ However, compared to gene editing, DNA methylation modulation in CAR‐T engineering is less intuitive and generally less effective, limiting its broader application. Nevertheless, clinical studies have demonstrated that dual‐drug (DP) therapy, combining DNA demethylating agents, achieves high response rates in relapsed/refractory Hodgkin's lymphoma^[^ ^93^, ^94^ ^]^ suggesting that despite its limitations, DNA methylation remains a relevant area of CAR‐T research. In contrast, chromatin accessibility offers broader and more practical applications for CAR‐T engineering. Chromatin accessibility undergoes dynamic changes during CAR‐T cell differentiation and exhaustion. ATAC‐seq enables the identification of key genes and regulatory factors such as LDHA,^[^ ^95^ ^]^ c‐Jun,^[^ ^96^ ^]^ TET2,^[^ ^97^ ^]^ FOXO1,^[^ ^7^, ^98^ ^]^ NR4A,^[^ ^99^ ^]^ BATF, and IRF4.^[^ ^100^ ^]^ This method provides insights into chromatin remodeling by tracking natural changes in accessibility or by assessing the impact of gene knockouts on chromatin structure. Although ATAC‐seq offers valuable information on chromatin accessibility, it is often complemented by ChIP‐seq or CUT&Tag to precisely map transcription factor‐binding sites and histone acetylation states. For example, ChIP‐seq revealed a cooperative interaction between BATF and IRF4, which collectively counteracts T‐cell exhaustion.^[^ ^101^ ^]^ Additionally, CUT&Tag has addressed some of the limitations of ChIP‐seq, such as the low signal‐to‐noise ratio and high input requirements. A recent CUT&T Tag study demonstrated that H3K27ac modifications promoted the formation of memory CAR‐T cells, significantly enhancing their antitumor potency.^[^ ^102^ ^]^ to chromatin accessibility, chromatin conformation is another promising research topic. Hi‐C analysis has shown that the 3D structure of chromatin influences T‐cell differentiation^[^ ^103^, ^104^, ^105^ ^]^ offering valuable insights into CAR‐T biology.

Compared to low‐throughput epigenomic profiling, RNA sequencing provides a comprehensive view of CAR‐T cell differentiation by capturing gene expression patterns in large cell populations. Intrinsic variability in autologous T cells is a key factor affecting CAR‐T cell therapy outcomes. A study analyzing pre‐infusion T cells from 71 patients using RNA‐seq identified TCF7 and LEF1 as critical regulators of early memory T cell states, whereas chronic interferon signaling was associated with poor CAR‐T cell persistence in clinical settings.^[^ ^106^ ^]^ To date, this is the most extensive molecular annotation of clinical CAR‐T therapy. Single‐cell proteomics complements transcriptomic analysis by profiling the surface proteins that define CAR‐T differentiation states. A CyTOF study demonstrated that CAR^+^ and CAR‐T cells exhibit distinct differentiation trajectories^[^ ^107^ ^]^ and that asymmetric distribution of surface proteins occurs during CAR‐T differentiation.^[^ ^108^ ^]^ Another CyTOF‐based immunophenotyping study revealed that pembrolizumab enhanced CAR‐T cell activation and proliferation.^[^ ^109^ ^]^ These omics approaches, which leverage different technologies, collectively provide a multidimensional perspective on CAR‐T cell differentiation and offer insights into strategies to mitigate exhaustion.

scRNA‐seq is a key technique used for subpopulation classification and lineage tracing.^[^ ^110^ ^]^ It enables the precise tracking of CAR‐T clonal subtypes^[^ ^111^, ^112^, ^113^ ^]^ and monitoring of their dynamic proportions over time.^[^ ^114^ ^]^ Notably, single‐cell transcriptomic profiling of bystander CAR‐T cells (CARneg T) suggests that they may possess intrinsic tumor‐killing capabilities^[^ ^115^ ^]^ and may contribute to cytokine release syndrome (CRS).^[^ ^116^, ^117^, ^118^ ^]^ However, single‐omics approaches alone are insufficient to fully capture the complexity of lineage relationships. Multi‐omics integration enhances our understanding of the functional heterogeneity across CAR‐T subpopulations, supporting the development of a more robust and diverse CAR‐T ecosystem. Studies combining scRNA‐seq and single‐cell T‐cell receptor sequencing (TCR‐seq) have identified distinct CAR‐T subsets with long‐term persistence and effective antitumor responses in patients with ALL.^[^ ^119^ ^]^ Furthermore, integrated analyses have revealed the presence of regulatory CAR‐T (CAR‐TREG) cells in commercial CAR‐T products, such as axicabtagene ciloleucel (axi‐cel) and tisagenlecleucel (tisa‐cel), which may suppress CAR‐T function and contribute to relapse.^[^ ^120^ ^]^ Longitudinal studies using TCR‐seq and multi‐omics have further characterized CAR‐T cell subsets, functionality, and molecular signatures in two patients over the last decade, providing valuable insights into the long‐term dynamics of CAR‐T cells.^[^ ^121^ ^]^ Proteomic, transcriptomic, and epigenomic analyses conducted by the National Institute of Health revealed that patients with robust in vivo expansion of GD2 CAR‐T cells had higher baseline levels of naïve T cells and CXCR3^+^ monocytes.^[^ ^122^ ^]^ Similarly, a multi‐omics study at Yale University identified type 2 helper T cells (Th2) as crucial for long‐term CAR‐T remission.^[^ ^123^ ^]^ A recent single‐cell multi‐omics study further demonstrated that type 2 CAR‐T cells maintain T‐cell homeostasis and promote sustained remission.^[^ ^124^ ^]^ Researchers have combined time‐lapse imaging microscopy with scRNA‐seq to identify, for the first time, a multifunctional CD8 T cell subset, termed CD8‐fit T cells.^[^ ^125^ ^]^ These T cells demonstrate a robust migratory capacity and prolonged cytotoxic activity. These findings provide critical guidance for engineering functionally diverse CAR T‐cell ecosystems.

Although lineage tracing is commonly used in longitudinal studies, recent research has underscored the importance of cross‐disease comparisons in defining CAR‐T functionality. A single‐cell multi‐omics study found that, compared to hematologic malignancies, CAR‐T subsets from patients with neuromyelitis optica spectrum disorder exhibited distinct cytotoxic suppression profiles.^[^ ^126^ ^]^ Another study integrating scRNA‐seq, TCR‐seq, and BCR‐seq characterized CAR‐T cell populations in patients with immune‐mediated necrotizing myopathy.^[^ ^127^ ^]^ These findings suggest that expanding CAR‐T lineage tracing beyond oncology may reveal novel insights into CAR‐T cell biology and broaden therapeutic applications.

### 2.3 Transitioning CAR‐T Cell Properties to Tumor Microenvironment and Immune Reconstruction

Increasing evidence suggests that the tumor microenvironment plays a pivotal role in determining the efficacy of CAR‐T cell therapy. The TME not only influences the infiltration capacity of CAR‐T cells, but also weakens their cytotoxic effects through immunosuppressive molecules, metabolic barriers, and other inhibitory mechanisms. Therefore, focusing solely on CAR‐T cells is insufficient to fully explain therapeutic outcomes. Therefore, a comprehensive understanding of the TME and its interactions with CAR‐T cells has emerged as a critical research direction.

Multi‐omics technologies provide multidimensional insights into the cellular composition and spatial heterogeneity of the TME, offering a more comprehensive perspective on their role in shaping CAR‐T cell responses (Figure 2c). A study integrating WGS, scRNA‐seq, ATAC‐seq, and TCR‐seq revealed that CD16⁺ macrophages within the TME of multiple myeloma patients treated with CD14⁺ CAR‐T cells exhibited an exhausted phenotype.^[^ ^128^ ^]^ Another study demonstrated that monocyte‐derived macrophages tend to adopt a pro‐tumor phenotype, which serves as a key driver of tumor relapse.^[^ ^129^ ^]^ However, conventional and single‐cell sequencing techniques disrupt the spatial architecture of cells during sample processing, limiting investigations into the spatial heterogeneity of the TME. The advent of spatial omics has overcome this limitation, enabling in situ analysis of the TME composition and dynamic changes. For instance, a study integrating spatial multi‐omics analyzed the TME of four patients with primary central nervous system lymphoma (PCNSL) patients, reconstructed the trajectory of cold‐to‐hot tumor transitions, and identified key transitional molecules.^[^ ^130^ ^]^ Additionally, we mapped the spatial distribution of CAR‐T cell targets to provide critical insights into the challenges of CAR‐T cell infiltration. Another spatial transcriptomic study revealed that specific cancer‐associated fibroblast subpopulations create an immune‐evading barrier by impeding CD8 + T‐cell infiltration into solid tumors.^[^ ^131^ ^]^ However, current spatial transcriptomic technologies lack TCR/BCR sequencing data, limiting the study of interactions between CAR‐T cells and endogenous immune components. To address this, researchers have developed a Spatial VDJ technique that enables spatial mapping of specific T cell clones across different tumor regions,^[^ ^132^ ^]^ offering new avenues for optimizing CAR‐T therapies.

The spatial distribution of drugs and metabolites within the TME is a critical research topic. For example, Zhang et al. demonstrated that the expression of glyceraldehyde‐3‐phosphate dehydrogenase and serine metabolism promote endothelial cell growth within the TME, thereby influencing CAR‐T cell infiltration.^[^ ^133^ ^]^ Spatial metabolomics provides deeper insight into how drugs regulate tumor metabolism.^[^ ^134^, ^135^ ^]^ Increasing evidence suggests that metabolic states play a pivotal role in determining T cell fate,^[^ ^136^ ^]^ offering a theoretical foundation for reprogramming CAR‐T cell metabolism to modulate differentiation trajectories.

Reconstruction of the endogenous T cell landscape following complete remission (CR) is another key determinant of CAR T cell therapy outcomes. Multi‐omics analyses have shown that after CD7⁺ T cell exhaustion, regenerated CD7⁻ T cells exhibit enhanced immune functionality compared to normal T cells, resembling immune characteristics observed in autoimmune diseases.^[^ ^137^, ^138^ ^]^ In pediatric patients with brain tumors receiving CAR‐T therapy, scRNA‐seq analysis of peripheral blood T cells revealed post‐treatment infiltration into the cerebrospinal fluid, accompanied by clonal expansion, indicating a response of the endogenous immune system to CAR‐T cells.^[^ ^139^ ^]^

### 2.4 Integrating Cross‐Organ Multi‐Omics for Toxicity Identification and Prognosis Prediction

In CAR‐T cell therapy, integrating multi‐omics data across human organs facilitates the identification of potential toxicity mechanisms and provides novel biomarkers for predicting prognosis (Figure 2d).

Some tumor antigens are expressed in rare cell subpopulations within specific tissues, potentially leading to adverse reactions following CAR‐T cell infusion, a phenomenon that is particularly evident in solid tumors.^[^ ^140^ ^]^ Given that these rare subpopulations often exhibit unique molecular characteristics, single‐omics approaches may struggle to accurately capture their variations. Consequently, multi‐omics integration plays a critical role in uncovering the potential toxicity mechanisms. For example, Van Oekelen et al. reported a case of a patient with MM who developed motor dysfunction after receiving BCMA CAR‐T therapy, with single‐cell data confirming BCMA expression in the caudate nucleus, suggesting potential neurotoxicity.^[^ ^141^ ^]^ Additionally, MacKay M et al. utilized multi‐omics data from 78 tissues, 124 cell types, and 20 cancer types to construct a target landscape for CAR‐T therapy, aiming to refine target selection, improve personalized patient matching, and minimize off‐target toxicity.^[^ ^142^ ^]^

CRS and immune effector cell‐associated neurotoxicity syndrome (ICANS) are common adverse events of CAR‐T therapy. Single‐cell sequencing indicated that the diversity of anti‐CD19 CAR T‐cell subpopulations was associated with the incidence of CRS and ICANS.^[^ ^143^ ^]^ Another study leveraging single‐cell multi‐omics analysis of microglia in patients with CRS revealed that their activation is primarily mediated by TAK1, suggesting that targeting TAK1 could serve as a potential therapeutic strategy for mitigating CAR‐T cell‐associated neurotoxicity.^[^ ^144^ ^]^ Additionally, single‐cell sequencing has revealed the risk of herpes virus reactivation following CAR‐T therapy.^[^ ^145^ ^]^ Notably, autoantibodies play a crucial role in the pathogenesis of autoimmune diseases, and PhIP‐Seq, an innovative technology, significantly enhances the resolution of autoantibody profiling. Recent findings suggest that BCMA CAR‐T therapy induces substantial alterations in the autoreactive antibody repertoire, whereas CD19/CD20 CAR‐T therapies exert relatively minor effects.^[^ ^146^ ^]^ These insights may pave the way for the application of CAR‐T cells in refractory autoimmune diseases.

Beyond toxicity assessment, multi‐omics integration has great potential for prognostic prediction of CAR‐T therapy. By analyzing biomarkers across various molecular layers, treatment responses can be assessed more precisely and long‐term clinical outcomes can be predicted. At the genomic level, CAPP‐seq enables the detection of circulating tumor DNA (ctDNA), providing prognostic insights for patients with relapsed/refractory LBCL undergoing CAR‐T therapy.^[^ ^147^ ^]^ In proteomics, IsoPlexis technology, based on microfluidic chips, allows for the assessment of cytokine and chemokine secretion by CAR‐T cells, facilitating the early prediction of toxicity events.^[^ ^148^ ^]^ Additionally, plasma proteomics has identified prognostic biomarkers for CAR‐T therapy, including disease‐specific proteins associated with ALL^[^ ^124^, ^149^ ^]^ and non‐Hodgkin's lymphoma (NHL)^[^ ^150^, ^151^ ^]^ Olink plasma proteomics has further revealed biomarkers linked to CAR‐T‐associated neurotoxicity.^[^ ^141^, ^152^ ^]^

Metabolomics also contributes significantly to the evaluation of CAR‐T cell prognosis. In a study analyzing plasma samples from 17 patients with MM receiving CAR‐T therapy, researchers identified LPCAT1 as a potential metabolic target in MM.^[^ ^153^ ^]^ Moreover, another study involving 20 patients with B‐ALL identified phosphatidylethanolamine, carnitine C12:1, and 1‐methylguanine as metabolic biomarkers that distinguished CR from non‐CR cases, highlighting the predictive value of metabolomics in CAR‐T therapy outcomes.^[^ ^149^ ^]^

Retrospective studies have suggested that gut microbiota may influence the efficacy and toxicity of CAR‐T therapy. Smith et al. found that members of the genera *Ruminococcus* and *Faecalibacterium* were associated with complete remission 100 post‐anti‐CD19 CAR‐T therapy.^[^ ^154^ ^]^ Additionally, Viktoria et al. demonstrated that a high abundance of *Dorea* and *Ruminococcaceae* correlated with CAR‐T cell expansion, immune checkpoint expression, and therapeutic efficacy in DLBCL patients receiving CD19‐directed CAR‐T cells.^[^ ^155^ ^]^ Hu et al. observed that severe CRS was linked to reduced *Bifidobacterium* abundance and post‐infusion microbiota diversity loss, accompanied by increased *Enterococcus* and *Actinomyces*.^[^ ^156^ ^]^ CAR‐T patients frequently receive antibiotics to mitigate infection risks; however, recent findings suggest that the presence of *Bacteroides, Ruminococcus, Alistipes*, and *Akkermansia* in patients with lymphoma can help predict treatment outcomes, provided that their microbiome remains intact and unaltered by broad‐spectrum antibiotics.^[^ ^157^ ^]^ These findings indicate that pre‐infusion 16S rRNA sequencing may be valuable in patient management strategies. Although recent research has revealed the presence of bacteria in various solid tumors^[^ ^158^ ^]^ the effect of the intratumoral microbiome on CAR‐T cell therapy remains largely unexplored.

## 3 Visualization Technology as a Critical Link Between Micro and Macro Scales in CAR‐T Therapy

As previously discussed, multiomics technologies play a critical role in the optimization of CAR‐T cell structures, cell lineage tracking, identification of specific subpopulations, tumor microenvironment research, toxicity assessment, and treatment prognosis prediction. However, multiomics approaches only provide static molecular data at specific time points, making it difficult to capture the 3D structure of CAR‐T cells and their real‐time dynamic changes. Therefore, they lack the ability to monitor the CAR‐T cell performance in a timely and intuitive manner. Visualization technologies address this limitation by bridging the gap between the microscopic and macroscopic levels. At the microscopic level, visualization technologies support the generation and validation of multiomics data, enabling researchers to directly observe molecular and cellular dynamics. At the macroscopic level, these technologies range from preclinical 3D‐printed tumor models that help assess CAR‐T cell infiltration and treatment efficacy to advanced microscopy imaging, which provides detailed insights into the fine structure and dynamic behavior of CAR‐T cells, and finally, to radiomics and in vivo imaging, which allows noninvasive, whole‐body tracking of CAR‐T cell dynamics in clinical settings. Thus, visualization technologies act as bridges for CAR‐T therapy, facilitating the transition from micro‐to macro‐perspectives, and advancing the evolution of 4D and 5D imaging concepts in CAR‐T treatment. The following sections discuss the latest research on various cutting‐edge visualization technologies that optimize CAR‐T therapies at both the preclinical and clinical stages (**Table** **2**).

**Table 2:** Research focuses of visualization techniques in CAR‐T therapy.

Visualization technique	Subfield	Research focus in CAR‐T therapy	Advantages	Disadvantages
**3D Printing**	3D tumor models	Evaluating CAR‐T cell infiltration into tumor tissues	Customizable patient‐specific tumor models	Incomplete recapitulation of the tumor microenvironment
	3D organoid models	Analyzing behavioral heterogeneity in CAR‐T and tumor organoid interactions	Cost‐effective platform for preclinical studies	Limited to early‐stage research applications
**Microscopy**	Super‐resolution microscopy	Identifying tumor cells with low antigen expression	High resolution; suitable for live‐cell imaging	High cost, time‐consuming; risk of phototoxicity
	Cryo‐electron microscopy	Guiding the design of single‐chain antibodies	High resolution; no staining required	Complex sample preparation; requires expensive instrumentation
	Transmission electron microscopy	Observing organelles within CAR‐T cells	High resolution; detailed ultrastructural analysis	Sample fragility; labor‐intensive preparation
	Confocal microscopy	Investigating CAR‐T cell interactions and immune synapse formation	Enables 3D imaging and multi‐channel detection	High phototoxicity; relatively slow imaging speed
	Integrated upright and inverted fluorescence microscopy	Visualizing CAR‐T infiltration into solid tumors	Broad applicability across cell types	Limited imaging depth
	Light sheet microscopy	Visualizing the 3D tumor microenvironment and dynamic CAR‐T cell‐mediated tumor killing	Fast 3D imaging; low phototoxicity	Complex preparation; high equipment costs
	Lattice light sheet microscopy	Real‐time imaging of CAR‐T cytotoxic processes at high spatiotemporal resolution	Ultra‐high temporal resolution; minimal phototoxicity	Technically complex; expensive
**Radiomics**	Bioluminescence imaging	In vivo tracking of CAR‐T cells using fluorescence	Non‐invasive monitoring	Limited to preclinical use
	PET/CT/MRI	Assessing clinical efficacy and detecting minimal residual disease	Longitudinal monitoring; early relapse detection	Radiation exposure; high cost; limited spatial resolution
	In vivo imaging	Monitoring tumor recurrence and tracking CAR‐T cells via radiolabeled probes or reporter genes	Real‐time, direct visualization of CAR‐T dynamics	Technically demanding; limited clinical translation

CAR‐T: chimeric antigen receptor t‐cell; 3D: three‐dimensional; PET: positron emission tomography; CT: computed tomography; MRI: magnetic resonance imaging.

### 3.1 3D‐Printed Solid Tumor Models for Evaluating CAR‐T Infiltration Capabilities

**Figure 3:**
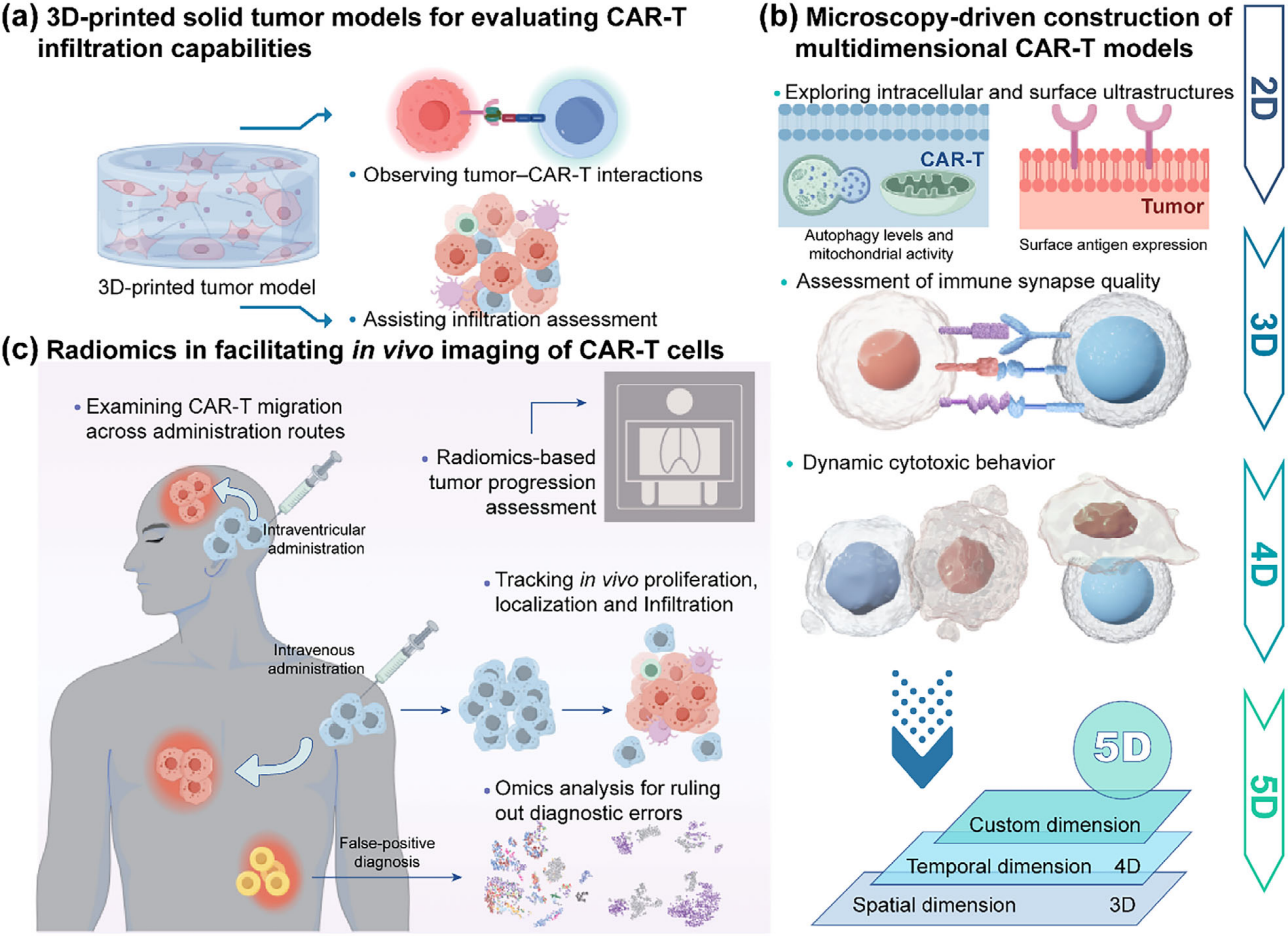
Visualization techniques bridging micro and macro research in CAR‐T therapy. a) 3D‐printed solid tumor models for assessing CAR‐T infiltration capacity. b) microscopy reveals intracellular and extracellular ultrastructures, immune synapses, and dynamic intercellular interactions, contributing to the construction of CAR‐T models in 3D, 4D, 5D, and beyond. c) in vivo imaging tracks CAR‐T expansion and localization, aiding in the evaluation of tumor progression and the impact of different administration routes.

In preclinical research, 3D printing technology has been used to construct tumor microenvironment models for solid tumors^[^ ^159^, ^160^, ^161^ ^]^ allowing researchers to visually observe the internal structure of tumors and assess the interaction between tumors and CAR‐T cells (**Figure** **3**a). One study investigated the CAR‐T cell‐induced cytotoxicity in a vascularized and dynamic breast tumor microenvironment for the first time.^[^ ^162^ ^]^ Another study created 3D glioblastoma models with varying degrees of stiffness. These models effectively replicated the mechanical properties of tumors and highlighted the infiltration and treatment challenges faced by CAR‐T cells in solid tumor environments.^[^ ^163^ ^]^ Furthermore, analyzing the behavioral heterogeneity of interactions between tumor organoids and CAR‐T cells is crucial.^[^ ^164^, ^165^, ^166^, ^167^ ^]^ The powerful combination of bio‐3D printing and organoids will undoubtedly advance our understanding of CAR‐T cell behavior.

### 3.2 Microscopy‐Driven Construction of Multidimensional CAR‐T Models (3D, 4D, and Beyond)

Although 3D printing provides a spatial and mechanical simulation of the tumor microenvironment, it remains insufficient for deciphering the molecular and subcellular structures and behavior of CAR‐T cells. Therefore, microscopic imaging technologies have become indispensable. Advancements in imaging within CAR T‐cell therapy have been driven by the interdisciplinary convergence of medical research and engineering innovations. This synergy has led to the development of cutting‐edge imaging techniques that enable researchers to precisely analyze the surface and internal 3D ultrastructures of CAR‐T cells, capture their 4D spatiotemporal dynamics, and incorporate additional biological parameters into 5D modeling (Figure 3b).

Super‐resolution microscopy, with its superior resolution compared to traditional optical methods, plays a crucial role in detecting CAR‐T cells at low antigen levels. Studies have shown that while flow cytometry struggles to detect CD19 in myeloma cells, super‐resolution microscopy can visualize low‐density CD19 molecules on the cell surface, highlighting its diagnostic potential.^[^ ^168^ ^]^ Cryo‐electron microscopy (cryo‐EM) offers structural insights into biomolecules, aiding the development of scFvs, as seen in the screening of a scFv that forms a stable complex with a myeloma‐specific antigen.^[^ ^169^ ^]^ Transmission electron microscopy (TEM), which visualizes intracellular organelles, such as autophagosomes and mitochondria, is critical for evaluating CAR‐T cell function. For example, autophagy inhibitors can enhance the cytotoxicity of CD19 CAR‐T cells^[^ ^170^ ^]^ and TEM can reveal the potential mitochondrial transfer between CAR‐T cells and tumor cells.^[^ ^171^ ^]^

Confocal microscopy enables the study of interactions between CAR‐T cells and between CAR‐T cells and tumor cells. Some researchers have designed CAR‐T cells with a chimeric switch receptor targeting PD‐L1 (CARP‐T), and confocal microscopy has revealed the interaction between CARP‐T cells and anti‐mesothelin CAR‐T cells, which enhances their anti‐tumor effects.^[^ ^172^ ^]^ Moreover, confocal microscopy has shown that CAR‐cytotoxic T lymphocyte (CTL) synapses lack distinct lymphocyte function‐associated antigen‐1 adhesion rings, exhibit disorganized tyrosine kinase clustering, and display rapid but less durable killing effects.^[^ ^173^ ^]^ Using confocal microscopy, Xiong et al. discovered that the immunological synapse quality of 4‐1BB‐CAR‐T cells was superior to that of CD28‐CAR‐T cells. In contrast, conventional methods, such as the standard ^51^Cr release assay and cytokine secretion assays, cannot distinguish between the two.^[^ ^174^ ^]^ As technology evolves, microscopic imaging may become as influential and convincing as the conventional techniques for CAR‐T therapy.

3D imaging technologies, such as light‐sheet fluorescence microscopy, allow the construction of immune microenvironment landscapes around CAR‐T cells. Real‐time image processing and z‐axis scanning enable fast imaging while minimizing phototoxicity and photobleaching. Recent innovations in 3D tissue pathology detection offer panoramic visualization and spatial analysis of tumor microenvironments with promising applications in CAR‐T research.^[^ ^175^ ^]^ In addition, integrated upright and inverted fluorescence microscopy demonstrated that nanoparticles loaded with CAR‐T cells exhibited stronger glioblastoma infiltration.^[^ ^176^ ^]^

With the evolution of technology, microscopy has expanded from 3D to 4D imaging, capturing both spatial and temporal dynamics. Innovations, such as Bessel oblique‐plane microscopy, enable real‐time 3D imaging of CAR‐T cytotoxic phenotypes, helping to quantify CAR‐T function.^[^ ^177^ ^]^ Lattice light‐sheet microscopy further enhances resolution, enabling detailed 4D models of CTL immunological synapse formation.^[^ ^178^ ^]^ Moving toward 5D imaging, researchers are constructing models that increasingly mirror the true microscopic environment, though opinions on what constitutes the fifth dimension vary.

### 3.3 Radiomics in Facilitating In Vivo Imaging of CAR‐T Cells

Although microscopic imaging techniques are primarily used for in vitro or tissue‐level studies, they are not well suited for real‐time monitoring of CAR‐T cells in patients. Therefore, in clinical settings, radiological imaging technologies have emerged as essential tools for tracking CAR‐T cell distribution in vivo, providing critical insights to guide clinical decision‐making (Figure 3c). Radiomics technologies, including computed tomography (CT), magnetic resonance imaging (MRI), and positron emission tomography (PET), significantly enhance the assessment of the clinical efficacy and depth of minimal residual disease (MRD) detection in CAR‐T cell therapy. Although bioluminescence imaging enables in vivo tracking of CAR‐T cells, it remains predominantly a preclinical method.^[^ ^179^ ^]^

PET typically detects tumor lesions based on metabolic activity, which can sometimes lead to false‐positive diagnoses. Multi‐omics sequencing has the potential to elucidate the underlying causes. Leipold et al. used PET imaging to evaluate patients with suspected myeloma relapse. Single‐cell sequencing of tissues from high FDG uptake regions in the lung revealed a population of TH17 cells responsible for the false‐positive diagnosis.^[^ ^180^ ^]^ PET/CT combines the high sensitivity of PET with the high spatial resolution of CT and is used to monitor long‐term remission following CAR‐T therapy.^[^ ^15^, ^143^, ^181^, ^182^ ^]^ PET/MRI can also detect MRDs,^[^ ^9^ ^]^ and is particularly well‐suited for imaging soft tissues such as the brain, spinal cord, and muscles. MRI revealed a new right temporal lobe brain lesion in a 68‐year‐old female with refractory DLBCL following CAR‐T therapy.^[^ ^183^ ^]^ Subsequent analysis identified anti‐CD19 CAR‐T cells in the cerebrospinal fluid. For autoimmune diseases, such as refractory antisynthetase syndrome with progressive myositis, MRI can detect signs of myositis, reflecting clinical improvements following CAR‐T infusion.^[^ ^18^ ^]^ Radiomics holds promise for enhancing our understanding of disease characteristics and progression.

In vivo tracking of CAR‐T cells using reporter gene‐based PET imaging is considered one of the most promising tools. Despite challenges, such as persistence and immunogenicity, significant progress has been made in tracking CAR‐T cells using this method.^[^ ^184^, ^185^, ^186^, ^187^ ^]^ Additionally, some researchers have proposed antigen‐based, real‐time, and noninvasive imaging of CAR‐T cells.^[^ ^188^ ^]^ Recently, in vivo CAR‐T cell therapy has entered clinical trials.^[^ ^189^ ^]^ Compared to *ex vivo* CAR‐T cells, in vivo CAR‐T cells can save time and costs while avoiding lymphodepletion. Complementary in vivo imaging technologies are required to support this advancement. These technologies should be capable of assessing the success rate of viral vectors or lipid nanoparticles targeting and entering T cells as well as monitoring the in vivo localization and tumor‐killing efficacy of CAR‐T cells.

Although most clinical trials deliver CAR‐T cells via intravenous injection, other routes of administration may be more effective for certain solid tumors. For instance, a study comparing intravenous, intraventricular, and intratumoral delivery of CAR‐T cells in brain tumors found that intratumoral injections were the most effective.^[^ ^190^ ^]^ The delivery method affects CAR‐T cell migration and localization in vivo. In vivo imaging can illuminate CAR‐T cell dynamics using different delivery methods, helping to optimize clinical administration strategies.

**Figure 4:**
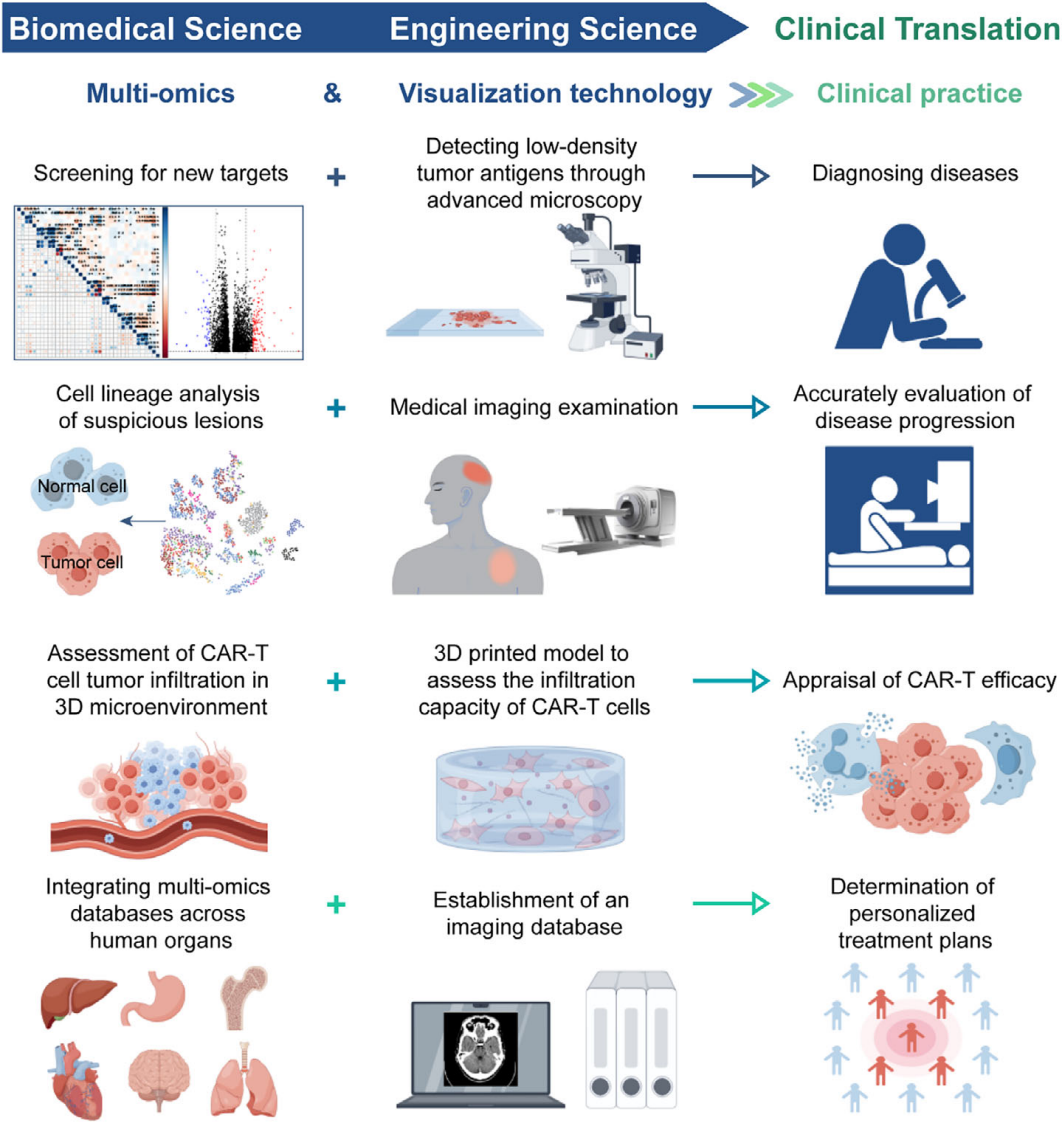
How to integrate multi‐omics and visualization technologies for clinical practice. The intersection of multi‐omics and visualization technologies in clinical practice is noteworthy. Multi‐omics enables the exploration of novel targets, while advanced microscopy techniques assess target density. Multi‐omics can also identify suspicious lesions observed in imaging studies. Both approaches evaluate CAR‐T cell tumor infiltration capabilities in different ways. Additionally, integrating both technologies into a unified database can provide personalized treatment plans for patients.

In summary, from 3D‐printed tumor models assessing CAR‐T cell infiltration to microscopic imaging characterizing CAR‐T cell function and behavior, and finally to medical imaging monitoring of treatment efficacy, visualization technologies provide critical insights into both mechanistic studies and clinical evaluation of CAR‐T therapy. Multi‐omics technologies enable the elucidation of the molecular regulatory mechanisms underlying CAR‐T cell function. The convergence of these two approaches facilitates a seamless transition from microscopic insights to macroscopic applications, driving CAR‐T therapy optimization (**Figure** **4**).

In target selection, multiomics analyses can identify novel tumor antigens, whereas advanced microscopic imaging techniques allow the detection of low‐abundance tumor antigens, improving the reliability of clinical diagnostics. In addition, PET imaging, which detects tumors based on metabolic activity, may produce false‐positive results. Multi‐omics sequencing can provide molecular‐level validation, reduce the misinterpretation of imaging data, and minimize diagnostic errors. To assess CAR‐T cell infiltration, single‐cell omics can reveal the adaptive responses of CAR‐T cells within the tumor microenvironment, whereas 3D‐printed models offer more intuitive experimental validation. Moreover, integrating multi‐organ omics data with imaging databases enables a comprehensive understanding of CAR‐T cell trafficking and target engagement in vivo, thereby providing valuable insights for personalized therapeutic strategies. Thus, by combining visualization technologies with multi‐omics analyses, researchers can not only enhance the mechanistic understanding of CAR‐T therapy but also improve its precise application in clinical settings.

## 4 AI‐Assisted Optimization of CAR‐T Therapy Across Multiple Dimensions

**Figure 5:**
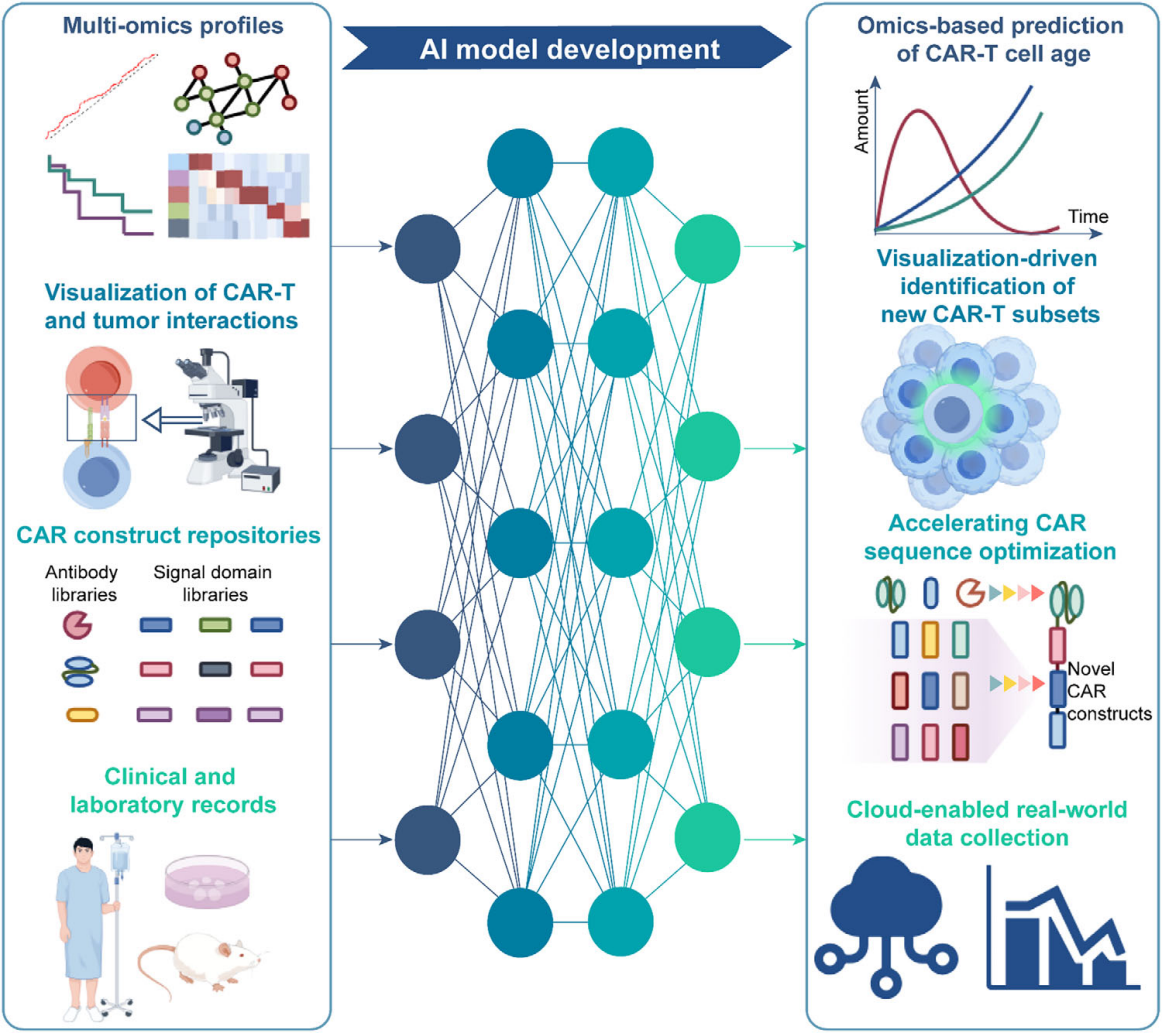
AI‐driven optimization of CAR‐T therapy. AI integrates multi‐omics, visualization, structural design, and clinical and laboratory data to enhance CAR‐T therapy. It aids in CAR‐T age prediction, novel subset identification, sequence optimization, and real‐world data collection, driving improvements in efficacy and clinical application.

CAR‐T therapy has entered an era of artificial intelligence, in which AI‐driven innovations are reshaping their design, monitoring, and clinical application**s** (**Figure** **5**). As a transformative force in the life sciences, AI leverages powerful data analysis, complex biological pattern recognition, and personalized medicine strategies to enhance every stage of CAR‐T development. From preclinical optimization to real‐world implementation, AI has provided unprecedented insights and accelerated advancements in CAR‐T therapy.

AI enhances the precision of multiomics analysis in CAR‐T research through deep learning and data mining. For example, in one study, researchers used single‐cell transcriptomics to cluster CD8 + T cells and applied machine learning to reveal age‐related transcriptional changes within subsets. This model demonstrates the ability to identify the developmental stage of infused CAR‐T cells, predict their cellular age and therapeutic outcomes, and assess treatment efficacy.^[^ ^191^ ^]^

AI can also refine the dynamic analysis of CAR‐T cell behavior by integrating it with visualization techniques. For instance, AI‐driven computer vision has been used to track thousands of interactions between CAR‐T and tumor cells during large B‐cell lymphoma treatment, leading to the identification of highly migratory and serially cytotoxic CAR‐T cell subpopulations.^[^ ^125^ ^]^ Additionally, deep learning algorithms can assist in analyzing rapidly evolving cellular dynamics, offering novel approaches for real‐time functional assessment of CAR‐T cells.^[^ ^35^ ^]^

Beyond multiomics and visualization, AI accelerates CAR‐T structural optimization through high‐throughput screening and rational design. AI‐powered molecular docking has shown promise for expediting the development of antibody fragments.^[^ ^192^, ^193^ ^]^ Building on this, advanced structure prediction models such as AlphaFold3^[^ ^194^ ^]^ have the potential to transform CAR design by accurately modeling CAR structures—an inherently challenging task due to the synthetic and non‐natural nature of CAR sequences. Conventional approaches often fall short in predicting the conformational landscapes of these engineered constructs, whereas AI‐driven methods provide a powerful alternative by generating high‐fidelity structural predictions. These computationally derived models can then be systematically evaluated using in silico tools to assess their stability, antigen‐binding properties, and functional performance, thereby streamlining CAR optimization and accelerating the development of next‐generation CAR‐T therapies. Furthermore, researchers have also developed an AI‐based CAR‐Toner platform that enables high‐throughput computation of charge patch indices.^[^ ^195^ ^]^ Importantly, this platform has been used to analyze the primary sequence types of CAR antigen‐binding domains, including single‐chain antibodies scFv, camelid nanobodies VHH, shark nanobodies VNAR, and variable lymphocyte receptor VLR sequences. These findings suggest that the camelid nanobody‐based CAR‐T design may offer clinical advantages. AI algorithms further optimize various CAR T‐cell domains, thereby expediting their development and refinement.^[^ ^196^ ^]^

Robotics has introduced new possibilities for CAR‐T cell therapy. Researchers engineered CAR‐T micro/nanorobots using metabolic click chemistry coupling, which enables active migration and infiltration into artificial tumor models under magnetoacoustic stimulation.^[^ ^197^ ^]^ This innovative strategy provides a new direction for precise CAR‐T delivery and potentially plays a critical role in cancer immunotherapy.

Although AI has contributed significantly to CAR‐T cell optimization at the preclinical stage, its clinical applications are equally promising. AI models can integrate multiomics data, imaging records, and experimental datasets to identify key biomarkers that influence CAR‐T cell efficacy and predict potential adverse events. Furthermore, cloud‐based platforms facilitate the collection and analysis of real‐world data, enabling AI to continuously refine CAR‐T‐cell therapy for improved efficacy and safety.

Moreover, the emergence of large language models (LLMs), such as GPT, DeepSeek, and Monkey,^[^ ^198^ ^]^ has further enhanced AI‐driven CAR‐T research and clinical applications. These models facilitate literature mining, hypothesis generation, and multi‐omics data interpretation. Clinically, they assist in extracting and structuring patient records, integrating multisource data, and identifying key features for predicting CAR‐T cell responses and adverse events. By integrating LLMs with multi‐omics and clinical data, AI can provide more comprehensive insights into CAR‐T cell optimization, ultimately improving treatment precision and patient outcomes. In summary, the diverse applications of AI technologies in CAR‐T therapy, along with their unique advantages, collectively drive the optimization and advancement of CAR‐T treatments (**Table** **3**).

**Table 3:** Summary of research directions and unique advantages of artificial intelligence in CAR‐T therapy.

Subfield	Research focus in CAR‐T therapy	Unique advantages
Machine Learning	Predicting CAR‐T cell differentiation	Analyzing complex datasets to uncover hidden patterns and make predictions
Computer Vision	Assisting in constructing 3D immune microenvironments	Accelerating visual data analysis from imaging technologies
Molecular Docking	CAR‐T antibody fragment development	Accelerating antibody development and selecting the optimal antibody
AI‐Driven Protein Structure Prediction	Predicting CAR 3D structure	Pre‐evaluating and screening different CAR structures to address structural challenges
Algorithmic platform	Optimizing CAR‐T structure	Enabling high‐throughput screening and accelerating development in CAR‐T structural optimization
Robotics	Engineering CAR‐T cell robots for therapeutic use	Enhancing the precision of CAR‐T therapy
Large Language Models (GPT, Deepseek, Monkey, and others)	Assisting in patient data integration and scientific knowledge synthesis	Enhancing clinical decision‐making and accelerating hypothesis generation

CAR‐T: chimeric antigen receptor T cell; 3D: three‐dimensional.

## 5 Conclusion and Future Prospect

In recent years, multiomics technologies have provided a rich array of tools for a deeper understanding of the molecular mechanisms underlying CAR‐T therapy.^[^ ^199^ ^]^ These technologies illuminate critical issues such as the persistence, toxicity, and tumor‐killing efficacy of CAR‐T cells, as well as tumor heterogeneity and the tumor microenvironment from various angles. Furthermore, the rapid progress in the interdisciplinary field of biomedical engineering, particularly in high‐end microscopy and in vivo imaging technologies, suggests that real‐time monitoring and dynamic adjustments of CAR‐T cell therapy will soon be within reach. Emerging technologies, such as nanotechnology, microelectronics, automated manufacturing, logic gating, and 3D cell scaffolds, have immense potential for modifying CAR‐T cells and facilitating their clinical translation.^[^ ^200^, ^201^, ^202^, ^203^, ^204^, ^205^ ^]^

Although significant progress has been made in multi‐omics and visualization technologies, conventional laboratory techniques and advanced artificial intelligence approaches remain invaluable. Cellular biology experiments and animal models form the bedrock upon which new technologies are built, and pathological assessments continue to serve as indispensable clinical tools. Additionally, techniques such as cytokine detection, polymerase chain reaction, and flow cytometry are commonly employed to evaluate the efficacy and persistence of CAR‐T therapy. Meanwhile, AI, with its powerful computational capabilities, has accelerated the exploration of CAR‐T mechanisms and the development of optimized CAR structures.

In the coming years, CAR‐T therapy is expected to expand its applications to a broader array of cancer types and explore novel therapeutic avenues in autoimmune diseases,^[^ ^206^, ^207^ ^]^ infectious diseases,^[^ ^208^, ^209^, ^210^ ^]^ and cardiac fibrosis.^[^ ^21^, ^211^ ^]^ Innovations such as multi‐targeted CAR‐T,^[^ ^212^, ^213^ ^]^ allogeneic CAR‐T,^[^ ^214^, ^215^, ^216^ ^]^ in vivo CAR‐T,^[^ ^217^ ^]^ personalized CAR‐T,^[^ ^218^ ^]^ CAR‐NK^[^ ^219^, ^220^ ^]^ and CAR‐Macrophage^[^ ^221^, ^222^ ^]^ et al are anticipated to enhance therapeutic efficacy, minimize side effects, and propel the advancement of immunotherapy. To overcome the current bottlenecks in these new CAR‐T cell products and advance the development of next‐generation CAR‐T cells, an integrated approach that leverages multiomics, advanced visualization, and emerging technologies is essential.

## Conflict of Interest

The authors declare no conflict of interest.

## Author Contributions

P.F. and Y.G. contributed equally to this work as co‐first authors. All authors have reviewed and endorsed the article. Y.G. and P.F. authored the manuscript and prepared the figures and tables. Y.Z., Y.X., and J.W. reviewed, revised, and contributed to the manuscript and the figures and tables.
